# Intratumor heterogeneity in HCC

**DOI:** 10.18632/aging.100760

**Published:** 2015-06-04

**Authors:** Juliane Friemel, Lukas Frick, Achim Weber

**Affiliations:** Institute of Surgical Pathology, University and University Hospital Zurich, 8091 Zurich, Switzerland

Hepatocellular carcinoma (HCC) is the second most common cause of cancer-related death worldwide [1], generally arising on the background of chronic liver diseases such as chronic viral hepatitis, alcohol-induced liver injury, or fatty liver disease. So far, classification proposals for HCC based on molecular markers are not yet routinely applied in surgical pathology or clinical management of HCC patients. This stands in contrast to the classification of hepatocellular adenoma (HCA), which has been included in the latest WHO classification [2], and is the basis for a stratified management of HCA patients.

Phenotypic intratumor heterogeneity in HCC with respect to morphology and differentiation grades within the same tumor is a well-known phenomenon to surgical pathologists. So far, genetic heterogeneity and the heterogeneity of biomarker expression in the surgical HCC specimens have not been systematically analyzed. However, to improve clinico-pathological classification systems and for the stratification of targeted therapies, it seems crucial to comprehensively characterize intratumor heterogeneity.

In a systematic analysis of 23 treatment-naïve unifocal HCC, we investigated individual tumors for morphologic, immunohistochemical and genetic intratumor heterogeneity as well as the association of these three features [3]. We found morphologic hetero-geneity in 87% of the tumors. Immunohistochemical heterogeneity with respect to five markers (CK7, CD44, AFP, EpCAM and glutamine synthethase) was present in 39% of cases and was always accompanied by morphologic heterogeneity. Clonal, i.e. genetic diversification was determined by sequencing the two most important HCC driver genes (*TP53* and *CTNNB1*). Combining Sanger sequencing with deep sequencing techniques facilitated the discovery of low frequency mutations and mitigated the effect of wild-type contamination. A mean of 7 regions per tumor was sequenced (120 areas in total), and genetic intratumor heterogeneity was found in 22% of cases. Thus, already analysis of the two main HCC driver genes clearly revealed that mutations are not homogeneously present in all regions of an individual tumor. This was found especially for *CTNNB1* mutations, but also for *TP53* mutations. The thorough dissection of morphologic, immunohistochemical and genomic intratumor hetero-geneity in our study illustrates that the primary co-existence of different growth patterns can be associated with divers biomarker expression and *TP53* or *CTNNB1* gene mutations among wild type tumor cells. Somatic mutations of various other genes are described in liver cancer, e.g. AXIN1 (WNT signaling), ARID and MLL genes (epigenetic modifiers), CDKN2A and IRF2 (cell cycle regulators interrelated with TP53) or TERT promoter mutations [4]. Although not comprehensively analyzed so far, it can be expected that the picture of subclonality within single liver lesions is even more complex. Tracking the expansion of certain tumor clones might also elucidate the development of multifocal liver cancer or disease progression favoring intra- and extrahepatic metastasis.

Intratumor heterogeneity has major implications for diagnosis and therapy of many solid cancers, indicating that a single tumor biopsy might not provide sufficient informative value regarding the molecular characteristics of the whole tumor. Primary renal cell cancer, as has been shown by Gerlinger et al. [5], displays common and private mutations throughout different, grossly demarcated tumor regions. Intratumor heterogeneity also limits the informative value of the widely used tissue microarray technique for studies on novel prognostic or predictive biomarkers.

The histopathologic and molecular classification of a tumor, often determined by the mutational status of a certain target, has therapeutic implications, e.g. in colorectal cancer (EGFR) or gastrointestinal stroma tumors (c-KIT). In the era of targeted therapies, intratumor heterogeneity is a major challenge to successful cancer therapy since it may result in primary resistance or early evasion of treatment to chemotherapeutic or molecular targeted substances. In melanoma, resistant tumor clones tend to evade chemotherapies and overgrow due to a forced selection process as well as an adaption process during tumor evolution. Therapy-induced inflammatory processes result not only in phenotypic plasticity of the tumor and its genomic landscape, but also alter the composition of tumor-associated immune cells. This recomposition of the microenviroment can contribute to cancer evolution and therapy resistance [6].

In conclusion, determining the degree of intratumor heterogeneity might be seen as a biomarker by itself and have prognostic value for disease progression [7]. With the high-throughput techniques widely available now, we envision the systematic investigation of intratumor heterogeneity in different types of cancer in order to pinpoint its clinical relevance. We expect that intratumor tumor heterogeneity with clinical relevance for molecular targeted therapy approaches will be present probably not in all, but in many tumor entities.

**Figure 1:**
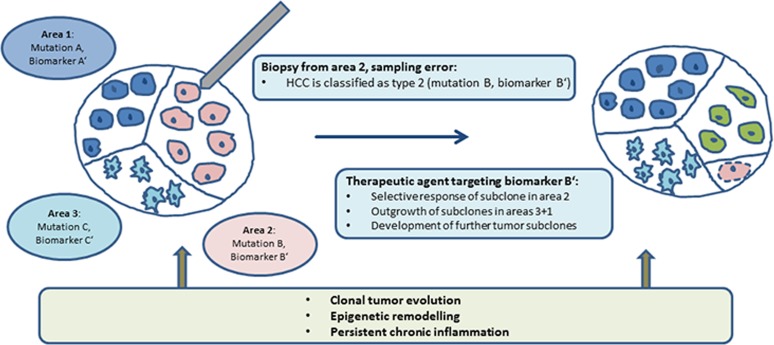
Implications of HCC intratumor heterogeneity for tumor classification and targeted therapy. A biopsy taken from tumor area 2 does not necessarily represent the whole tumor. This may result in a short falling tumor classification as type B, and potentially in an incomplete therapy response due to sensitivity of only one tumor subclone (mutation B, biomarker B‘, pink).

## References

[1] Ferlay J (2015). International journal of cancer.

[2] Bioulac-Sage P, B C., Wanless I. (2010). WHO classification of tumors of the digestive system.

[3] Friemel J (2015). Clinical cancer research: an official journal of the American Association for Cancer Research.

[4] Nault JC, Zucman-Rossi J (2014). Journal of hepatology.

[5] Gerlinger M (2012). The New England journal of medicine.

[6] Holzel M (2013). Nature reviews Cancer.

[7] Almendro V (2013). Annual review of pathology.

